# Chloroplast genome of *Tillandsia marconae* till & Vitek (Bromeliaceae), a hyperarid desert endangered species

**DOI:** 10.1080/23802359.2021.1944364

**Published:** 2021-08-08

**Authors:** Julio César Chávez-Galarza, Stefanny Cardenas-Ninasivincha, Roberto Contreras, Rubén Ferro-Mauricio, Wilson Huanca-Mamani

**Affiliations:** a Laboratorio de Investigación Tecnológica en Cambio Climático, Instituto Nacional de Innovación Agraria, Lima, Perú; b Departamento Académico de Ciencias Básicas y Afines, Universidad Nacional de Barranca, Barranca, Perú; c Laboratorio de Biología Molecular de Plantas, Facultad de Ciencias Agronómicas, Universidad de Tarapacá, Arica, Chile; d Centro Regional de Investigación y Desarrollo Sustentable de Atacama (CRIDESAT), Universidad de Atacama, Copiapó, Chile

**Keywords:** Chloroplast genome, *Tillandsia*

## Abstract

*Tillandsia marconae* Till & Vitek (Bromeliaceae) is a rare plant native species that grows over sand, in the coastal desert from Perú and Chile and is considered an endangered species. In this study, we assembled its chloroplast genome. The draft chloroplast genome of *T. marconae* is *ca*. 158,873 bp in length, containing a large single-copy region of 86,937 bp, a small single-copy region of 18,506 bp, and a pair of inverted repeat regions of 26,715 bp. The GC content of the draft chloroplast genome is 37.4%. It encodes a total of 135 genes, including 86 protein-coding genes, 38 tRNA genes, 8 rRNA genes, and three pseudogenes. The phylogenetic tree indicated that *T. marconae* is placed within the Bromeliaceae family and a close relationship with *Tillandsia usneoides* with 100% support.

*Tillandsia marconae* Till & Vitek is a sandy, perennial herbaceous and rare species with a very particular distribution. It is found naturally only in two separated locations in the coastal desert of South America; Marcona hill (Departamento de Ica) in Central Perú and Pampa Dos Cruces (Región de Arica y Parinacota) in northernmost of Chile, which is part of Atacama Desert (Rundel and Dillon 1998; Pinto et al. 2006). This species has evolved a highly specialized growth habit, unrooted on the sand, and use the regular fog humidity to survive in hyperarid coastal zones and also due to its small population, it is considered as endangered species in both countries (Zizka et al. 2009; Whaley et al. 2019; MMA 2021). These reasons make it necessary to implement conservation strategies and develop tools to study this rare species' ecological and evolutionary aspects. So far, the chloroplast genome of *Tillandsia usneoides* within the genus *Tillandsia* has been reported only (Poczai and Hyvönen 2017).

In this study, we reported the draft chloroplast genome sequence of *T. marconae* as an initial approach toward developing genomic tools to understands this species and its close relatives. The sample of *T. marconae* was collected from Pampa Dos Cruces, Arica, Chile (18°28′43.48″ S; 70°5′ 16.69″W). A specimen of *T. marconae* is deposited in the herbarium of Universidad de Concepción, Chile (http://www2.udec.cl/∼herbconc/, Dra. Alicia Marticorena, amartic@udec.cl) under the voucher CONC-156141. Total genomic DNA was extracted from fresh leaves using the DNeasy Plant Mini Kit (Qiagen, Hilden, Germany) following the manufacturer's instructions. DNA library was sequenced, and 150 pb paired-end were generated on an Illumina NovaSeq PE150 platform. After base quality control using FASTQC v01 (Andrews 2010), the remaining high-quality reads were used to assemble the chloroplast genome by SPAdes (Bankevich et al. 2012) and NOVOPlasty v01 (Dierckxsens et al. 2017), using *T. usneoides* chloroplast genome as a reference (KY293680). The chloroplast genome annotation was performed by GeSeq (Tillich et al. 2017).

The draft chloroplast genome sequence of *T. marconae* (GenBank accession number: MW415432) displays 158,873 bp in length with 37.4% CG content. It presents a quadripartite structure that contains a pair of inverted repeats (IR) regions (26,715 bp, GC content 42.7%), separated by a large single-copy (LSC) region (86,937 bp, GC content 35.3%) and a small single-copy (SSC) region (18,506 bp, GC content 31.5%). A total of 135 genes were predicted, including 38 tRNA genes (30 tRNA species), 8 rRNA genes (four rRNA species), 86 protein-coding genes (79 PCG species), and three pseudogenes (two pseudogene species).

To confirm the phylogenetic location of *T. marconae*, a maximum-likelihood tree was constructed with 1000 bootstrap replicates using IQ-Tree software (Nguyen et al. 2015) under GTR + I + G nucleotide substitution model, which was selected by Jmodeltest 2 (Darriba et al. 2012). The chloroplast genome of *T. marconae* was compared and aligned withother 18 chloroplast genomes obtained from GenBank by the MAFFT version 7.475 software (Katoh and Standley 2013), involving concatenation of 75 orthologue protein-coding genes. Four species from Bromeliaceae, three from Arecaceae, two from Zingiberaceae, and one each from Typhaceae, Eriocaulaceae, Musaceae, Liliaceae and Dioscoreaceae corresponding to Liliopsida class were included in the analysis, and with two species from Magnoliaceae, and one each from Piperaceae and Schisandraceae corresponding to Magnoliopsida class as outgroup. As expected, *T. marconae* is placed under the Bromeliaceae family and closest to *T. usneoides* (Figure 1). The published *T. marconae* chloroplast genome provides useful information into conservation, phylogenetic and evolutionary studies for this desert plant species.

**Figure 1.**
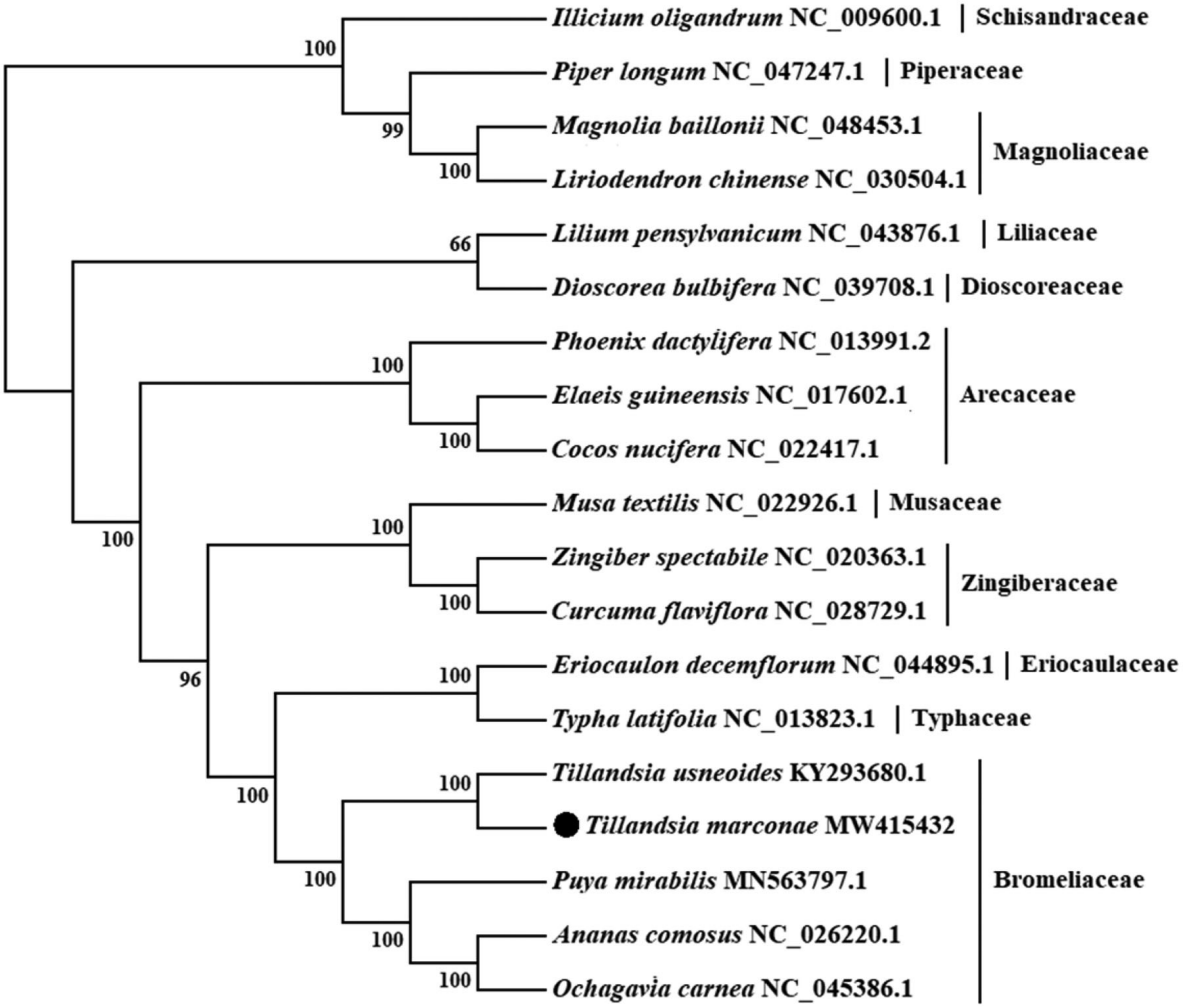
Maximum likelihood (ML) tree based on chloroplast genome sequences of 19 species. Values along branches correspond to ML bootstrap percentages.

## Data Availability

The data that supports this study is openly available in Genbank of NCBI (https://www.ncbi.nlm.nih.gov) under the accession number MW415432. The associated Bioproject, Biosample and SRA numbers are PRJNA687917, SAMN17159914, and SRR14267567, respectively.
